# Supplementary material for: Hot spot prediction in protein-protein interactions by an ensemble system
Source: BMC Syst Biol. 2018 Dec 31;12(Suppl 9):132. doi: 10.1186/s12918-018-0665-8 (PMC6311905; doi:10.1186/s12918-018-0665-8)
Supplement: Supplementary file 2 — All relevant MATLAB code. A simple Matlab implement of our predictor. (ZIP 19,742 kb) [file 12918_2018_665_MOESM2_ESM.zip › Additional file 2/Read ASA/ASA website/dbMPIKT/4U6HE.html]

 NetSurfP - Protein Surface Accessibility and Secondary Structure Predictions

  

|  |  |  |
| --- | --- | --- |
|  |  | NetSurfP - Protein Surface Accessibility and Secondary Structure Predictions  Technical University of Denmark |

  


---

```
# For publication of results, please cite:
# A generic method for assignment of reliability scores applied to solvent accessibility predictions.
# Bent Petersen, Thomas Nordahl Petersen, Pernille Andersen, Morten Nielsen and Claus Lundegaard
# BMC Structural Biology 2009, 9:51 doi:10.1186/1472-6807-9-51
#
# Column 1: Class assignment - B for buried or E for Exposed - Threshold: 25% exposure, but not based on RSA
# Column 2: Amino acid
# Column 3: Sequence name
# Column 4: Amino acid number
# Column 5: Relative Surface Accessibility - RSA
# Column 6: Absolute Surface Accessibility
# Column 7: Z-fit score for RSA prediction
# Column 8: Probability for Alpha-Helix
# Column 9: Probability for Beta-strand
# Column 10: Probability for Coil
E M  4U6HE                  1    0.777 155.398  -1.148   0.016   0.005   0.979
E G  4U6HE                  2    0.598  47.063  -1.293   0.257   0.016   0.727
E A  4U6HE                  3    0.494  54.439  -1.075   0.522   0.016   0.462
E A  4U6HE                  4    0.382  42.118  -0.758   0.660   0.049   0.291
E A  4U6HE                  5    0.421  46.438   0.085   0.751   0.050   0.199
E S  4U6HE                  6    0.337  39.532   0.394   0.831   0.044   0.125
B I  4U6HE                  7    0.080  14.874   0.035   0.911   0.033   0.057
E Q  4U6HE                  8    0.326  58.241   0.575   0.938   0.007   0.055
B T  4U6HE                  9    0.263  36.534   0.377   0.938   0.007   0.055
B T  4U6HE                 10    0.074  10.222   0.078   0.970   0.001   0.030
B V  4U6HE                 11    0.068  10.436   0.600   0.970   0.001   0.030
E N  4U6HE                 12    0.312  45.750  -0.118   0.970   0.001   0.030
B T  4U6HE                 13    0.203  28.087   1.031   0.988   0.000   0.012
B L  4U6HE                 14    0.042   7.690   0.261   0.988   0.000   0.012
B S  4U6HE                 15    0.082   9.646   0.214   0.988   0.000   0.012
E E  4U6HE                 16    0.429  75.034   0.791   0.988   0.000   0.012
E R  4U6HE                 17    0.347  79.394   1.611   0.988   0.000   0.012
B I  4U6HE                 18    0.053   9.805   0.715   0.988   0.000   0.012
B S  4U6HE                 19    0.145  16.982   0.092   0.970   0.001   0.030
E S  4U6HE                 20    0.501  58.682   0.177   0.923   0.002   0.076
E K  4U6HE                 21    0.320  65.886   1.290   0.938   0.007   0.055
B L  4U6HE                 22    0.053   9.668   0.720   0.879   0.010   0.111
E E  4U6HE                 23    0.354  61.809  -0.466   0.717   0.014   0.269
E Q  4U6HE                 24    0.406  72.422  -0.148   0.561   0.047   0.393
E E  4U6HE                 25    0.325  56.690  -0.284   0.502   0.102   0.396
B A  4U6HE                 26    0.196  21.610  -1.344   0.191   0.086   0.723
E N  4U6HE                 27    0.429  62.791  -0.838   0.113   0.087   0.800
B A  4U6HE                 28    0.275  30.327  -0.826   0.113   0.087   0.800
E S  4U6HE                 29    0.433  50.712  -1.113   0.118   0.150   0.732
B A  4U6HE                 30    0.278  30.669  -1.198   0.056   0.142   0.802
E Q  4U6HE                 31    0.383  68.493  -0.305   0.066   0.296   0.638
B T  4U6HE                 32    0.265  36.700  -0.967   0.069   0.386   0.545
E K  4U6HE                 33    0.436  89.665   0.349   0.021   0.451   0.528
B C  4U6HE                 34    0.054   7.554   0.161   0.022   0.552   0.426
E D  4U6HE                 35    0.288  41.544   0.203   0.004   0.616   0.381
B I  4U6HE                 36    0.059  10.989   0.612   0.002   0.816   0.182
E E  4U6HE                 37    0.287  50.191   0.606   0.001   0.900   0.099
B I  4U6HE                 38    0.078  14.467  -0.018   0.002   0.816   0.182
B G  4U6HE                 39    0.219  17.212  -1.026   0.004   0.616   0.381
E N  4U6HE                 40    0.311  45.530   0.180   0.003   0.718   0.279
B F  4U6HE                 41    0.075  14.992  -0.211   0.001   0.900   0.099
E Y  4U6HE                 42    0.334  71.376   0.676   0.001   0.900   0.099
B I  4U6HE                 43    0.251  46.343  -1.031   0.023   0.655   0.322
E R  4U6HE                 44    0.412  94.394  -0.717   0.004   0.420   0.576
E Q  4U6HE                 45    0.558  99.570   0.229   0.005   0.336   0.660
B N  4U6HE                 46    0.297  43.481  -1.373   0.005   0.262   0.733
E H  4U6HE                 47    0.562 102.282  -0.100   0.004   0.138   0.858
E G  4U6HE                 48    0.521  40.995   0.452   0.004   0.138   0.858
B C  4U6HE                 49    0.043   5.967  -0.465   0.004   0.514   0.481
E N  4U6HE                 50    0.397  58.150   1.606   0.001   0.900   0.099
B L  4U6HE                 51    0.049   9.045   0.989   0.006   0.962   0.032
E T  4U6HE                 52    0.388  53.857   1.617   0.006   0.962   0.032
B V  4U6HE                 53    0.061   9.453   0.344   0.006   0.962   0.032
E K  4U6HE                 54    0.277  57.041   1.124   0.006   0.962   0.032
B N  4U6HE                 55    0.145  21.287  -1.604   0.018   0.846   0.136
E M  4U6HE                 56    0.282  56.508   1.157   0.023   0.655   0.322
B C  4U6HE                 57    0.055   7.778  -0.776   0.022   0.359   0.619
E S  4U6HE                 58    0.362  42.462  -0.400   0.019   0.141   0.840
E A  4U6HE                 59    0.375  41.336  -1.088   0.058   0.017   0.925
E D  4U6HE                 60    0.452  65.205   0.011   0.115   0.016   0.868
E A  4U6HE                 61    0.318  35.044  -0.625   0.694   0.003   0.303
E D  4U6HE                 62    0.543  78.290   0.221   0.858   0.002   0.139
B A  4U6HE                 63    0.249  27.451   0.586   0.923   0.002   0.076
B Q  4U6HE                 64    0.118  21.146   0.239   0.923   0.002   0.076
B L  4U6HE                 65    0.054   9.924   0.993   0.970   0.001   0.030
E D  4U6HE                 66    0.403  58.144   1.624   0.988   0.000   0.012
B A  4U6HE                 67    0.103  11.384   0.694   0.988   0.000   0.012
B V  4U6HE                 68    0.028   4.273   0.736   0.988   0.000   0.012
B L  4U6HE                 69    0.083  15.216   0.606   0.988   0.000   0.012
E S  4U6HE                 70    0.450  52.799   1.332   0.988   0.000   0.012
B A  4U6HE                 71    0.061   6.766   0.244   0.988   0.000   0.012
B A  4U6HE                 72    0.036   3.912  -0.240   0.988   0.000   0.012
E T  4U6HE                 73    0.296  41.069   1.196   0.988   0.000   0.012
E E  4U6HE                 74    0.470  82.179   0.524   0.970   0.001   0.030
B T  4U6HE                 75    0.069   9.501   0.320   0.923   0.002   0.076
B Y  4U6HE                 76    0.140  29.875   0.234   0.858   0.002   0.139
E S  4U6HE                 77    0.651  76.285   0.892   0.600   0.003   0.397
E G  4U6HE                 78    0.427  33.573   0.214   0.246   0.004   0.750
B L  4U6HE                 79    0.064  11.755   0.655   0.109   0.005   0.886
E T  4U6HE                 80    0.375  52.026   1.430   0.053   0.005   0.942
E P  4U6HE                 81    0.639  90.717   0.933   0.858   0.002   0.139
E E  4U6HE                 82    0.705 123.251   0.876   0.858   0.002   0.139
B Q  4U6HE                 83    0.269  48.026   0.024   0.858   0.002   0.139
B K  4U6HE                 84    0.278  57.123   0.337   0.923   0.002   0.076
E A  4U6HE                 85    0.562  61.899   0.779   0.923   0.002   0.076
E Y  4U6HE                 86    0.375  80.052   0.816   0.923   0.002   0.076
B V  4U6HE                 87    0.037   5.718  -0.018   0.858   0.002   0.139
B P  4U6HE                 88    0.257  36.412  -0.584   0.782   0.003   0.216
E A  4U6HE                 89    0.623  68.688   0.103   0.717   0.014   0.269
B M  4U6HE                 90    0.289  57.909  -0.069   0.717   0.014   0.269
B F  4U6HE                 91    0.139  27.917   0.079   0.622   0.015   0.363
B T  4U6HE                 92    0.251  34.744  -1.010   0.561   0.047   0.393
B A  4U6HE                 93    0.242  26.690  -0.487   0.502   0.102   0.396
E A  4U6HE                 94    0.369  40.620  -1.090   0.538   0.173   0.289
B L  4U6HE                 95    0.196  35.906   0.310   0.428   0.171   0.402
E N  4U6HE                 96    0.554  81.120  -0.949   0.199   0.152   0.649
B I  4U6HE                 97    0.259  47.970  -0.044   0.113   0.087   0.800
E Q  4U6HE                 98    0.532  94.979  -0.620   0.118   0.150   0.732
E T  4U6HE                 99    0.440  61.014  -0.292   0.113   0.087   0.800
E S  4U6HE                100    0.647  75.793  -0.425   0.113   0.087   0.800
B V  4U6HE                101    0.151  23.147   0.288   0.113   0.087   0.800
E N  4U6HE                102    0.569  83.316  -0.088   0.184   0.043   0.773
E T  4U6HE                103    0.538  74.607   0.514   0.278   0.093   0.628
B V  4U6HE                104    0.117  17.952  -0.443   0.278   0.093   0.628
E V  4U6HE                105    0.293  44.988  -0.192   0.191   0.086   0.723
E R  4U6HE                106    0.596 136.461  -0.320   0.113   0.087   0.800
E D  4U6HE                107    0.658  94.847  -0.755   0.053   0.043   0.903
E F  4U6HE                108    0.259  51.941   0.043   0.113   0.043   0.844
E E  4U6HE                109    0.616 107.633   0.053   0.199   0.152   0.649
E N  4U6HE                110    0.429  62.820  -0.271   0.321   0.252   0.427
E Y  4U6HE                111    0.316  67.615   0.253   0.352   0.332   0.316
B V  4U6HE                112    0.141  21.687  -0.175   0.352   0.332   0.316
E K  4U6HE                113    0.420  86.291   0.850   0.373   0.401   0.226
E Q  4U6HE                114    0.466  83.263   0.440   0.252   0.423   0.325
E T  4U6HE                115    0.482  66.853   1.126   0.231   0.330   0.439
B C  4U6HE                116    0.059   8.340   0.001   0.066   0.296   0.638
E N  4U6HE                117    0.549  80.374   0.843   0.069   0.386   0.545
B S  4U6HE                118    0.223  26.112  -0.971   0.069   0.386   0.545
E S  4U6HE                119    0.498  58.389  -0.230   0.074   0.484   0.442
B A  4U6HE                120    0.169  18.580  -0.919   0.079   0.592   0.329
E V  4U6HE                121    0.391  60.066   0.000   0.074   0.484   0.442
B V  4U6HE                122    0.148  22.748   0.258   0.074   0.484   0.442
E D  4U6HE                123    0.530  76.344   0.083   0.069   0.386   0.545
E N  4U6HE                124    0.426  62.396  -0.238   0.021   0.279   0.699
E K  4U6HE                125    0.428  88.101   0.565   0.021   0.451   0.528
B L  4U6HE                126    0.107  19.518   0.808   0.023   0.655   0.322
E K  4U6HE                127    0.454  93.326   1.491   0.023   0.655   0.322
B I  4U6HE                128    0.102  18.888   0.619   0.004   0.616   0.381
E Q  4U6HE                129    0.451  80.477   0.347   0.004   0.616   0.381
E N  4U6HE                130    0.455  66.656   0.980   0.003   0.718   0.279
B V  4U6HE                131    0.089  13.756   1.283   0.018   0.846   0.136
E I  4U6HE                132    0.441  81.659   1.334   0.021   0.756   0.223
B I  4U6HE                133    0.104  19.295   0.125   0.022   0.552   0.426
E D  4U6HE                134    0.452  65.119  -0.847   0.021   0.279   0.699
E E  4U6HE                135    0.462  80.694   0.174   0.021   0.279   0.699
B C  4U6HE                136    0.063   8.789  -0.836   0.021   0.279   0.699
E Y  4U6HE                137    0.369  78.748  -0.657   0.019   0.141   0.840
B G  4U6HE                138    0.229  18.014  -1.209   0.005   0.045   0.951
E A  4U6HE                139    0.352  38.823  -0.202   0.005   0.015   0.979
E P  4U6HE                140    0.697  98.833  -1.015   0.003   0.003   0.994
E G  4U6HE                141    0.575  45.221  -2.076   0.005   0.015   0.979
E S  4U6HE                142    0.411  48.134   0.358   0.005   0.262   0.733
E P  4U6HE                143    0.320  45.365  -0.036   0.003   0.718   0.279
B T  4U6HE                144    0.064   8.918   0.563   0.001   0.900   0.099
E N  4U6HE                145    0.241  35.312   1.178   0.001   0.900   0.099
B L  4U6HE                146    0.035   6.463   0.371   0.001   0.959   0.040
B E  4U6HE                147    0.135  23.602   0.955   0.001   0.959   0.040
B F  4U6HE                148    0.049   9.834   0.337   0.001   0.959   0.040
B I  4U6HE                149    0.072  13.376   0.317   0.001   0.900   0.099
B N  4U6HE                150    0.111  16.265  -0.709   0.004   0.616   0.381
B T  4U6HE                151    0.195  27.033  -0.563   0.019   0.141   0.840
E G  4U6HE                152    0.339  26.679  -1.945   0.053   0.043   0.903
E S  4U6HE                153    0.327  38.313  -0.311   0.184   0.043   0.773
B S  4U6HE                154    0.166  19.467  -0.925   0.354   0.048   0.598
E K  4U6HE                155    0.341  70.226  -0.029   0.561   0.047   0.393
B G  4U6HE                156    0.103   8.098  -1.348   0.660   0.049   0.291
B N  4U6HE                157    0.200  29.207  -0.465   0.717   0.014   0.269
B C  4U6HE                158    0.052   7.259   0.165   0.802   0.014   0.185
B A  4U6HE                159    0.063   6.910  -0.468   0.938   0.007   0.055
B I  4U6HE                160    0.069  12.839  -0.031   0.970   0.001   0.030
E K  4U6HE                161    0.321  66.009   1.365   0.988   0.000   0.012
B A  4U6HE                162    0.130  14.348   0.452   0.988   0.000   0.012
B L  4U6HE                163    0.049   8.990  -0.006   0.988   0.000   0.012
B M  4U6HE                164    0.108  21.511   0.074   0.970   0.001   0.030
E Q  4U6HE                165    0.470  83.942   1.779   0.970   0.001   0.030
B L  4U6HE                166    0.063  11.590  -0.162   0.923   0.002   0.076
B T  4U6HE                167    0.177  24.536  -0.261   0.858   0.002   0.139
E T  4U6HE                168    0.442  61.347  -0.492   0.782   0.003   0.216
E K  4U6HE                169    0.488 100.464   0.683   0.600   0.003   0.397
B A  4U6HE                170    0.220  24.288  -1.490   0.430   0.016   0.555
E T  4U6HE                171    0.392  54.398  -0.595   0.257   0.016   0.727
E T  4U6HE                172    0.624  86.604  -0.138   0.191   0.086   0.723
E Q  4U6HE                173    0.554  99.016   0.994   0.135   0.317   0.548
B I  4U6HE                174    0.143  26.510  -0.378   0.069   0.386   0.545
E A  4U6HE                175    0.321  35.352  -0.009   0.064   0.216   0.721
E P  4U6HE                176    0.506  71.730  -1.305   0.056   0.142   0.802
E K  4U6HE                177    0.510 104.907  -0.342   0.113   0.087   0.800
E Q  4U6HE                178    0.369  65.921  -0.499   0.191   0.086   0.723
E V  4U6HE                179    0.460  70.733  -1.404   0.191   0.086   0.723
E A  4U6HE                180    0.571  62.946  -0.557   0.184   0.043   0.773
E G  4U6HE                181    0.528  41.577  -2.533   0.113   0.043   0.844
E T  4U6HE                182    0.577  79.974  -0.949   0.053   0.043   0.903
E G  4U6HE                183    0.710  55.877  -1.525   0.018   0.047   0.935
E V  4U6HE                184    0.622  95.601  -1.377   0.003   0.003   0.994

Explain the output.  Go back.
```

---
